# Mutation profile of *FLNC* gene and its prognostic relevance in patients with hypertrophic cardiomyopathy

**DOI:** 10.1002/mgg3.488

**Published:** 2018-11-08

**Authors:** Hao Cui, Jizheng Wang, Ce Zhang, Guixin Wu, Changsheng Zhu, Bing Tang, Yubao Zou, Xiaohong Huang, Rutai Hui, Lei Song, Shuiyun Wang

**Affiliations:** ^1^ Department of Cardiac Surgery Chinese Academy of Medical Sciences and Peking Union Medical College Beijing China; ^2^ State Key Laboratory of Cardiovascular Diseases Chinese Academy of Medical Sciences and Peking Union Medical College Beijing China; ^3^ Department of Cardiology Fuwai Hospital National Center for Cardiovascular Disease Chinese Academy of Medical Sciences and Peking Union Medical College Beijing China

**Keywords:** *FLNC* mutation, hypertrophic cardiomyopathy, penetrance, prognosis

## Abstract

**Background:**

Filamin C (*FLNC*) mutation was reported as a cause of HCM, with a high probability of sudden cardiac death. However, the mutation profile of *FLNC*, and its relationship with phenotypic expression in HCM, remains to be elucidated.

**Methods:**

In this study, *FLNC* gene was sequenced in 540 HCM patients and 307 healthy controls.

**Results:**

We found that 39 (7.2%) patients carried *FLNC* mutations, with a similar frequency to that of controls (4.2%, *p* = 0.101). Pedigree analysis showed that mutations were not well segregated with HCM. The baseline characteristics between HCM patients, with and without mutations, were comparable. *FLNC* mutations did not increase the risk for either all‐cause mortality (HR 0.746, 95% CI 0.222–2.295, *p* = 0.575) or cardiac mortality (HR 0.615, 95% CI 0.153–1.947, *p* = 0.354) in HCM patients during a follow‐up of 4.7 ± 3.2 years. Moreover, there was no significant difference in survival free from sudden cardiac arrest (HR 0.721, 95% CI 0.128–3.667, *p* = 0.660) and heart failure (HR 0.757, 95% CI 0.318–1.642, *p* = 0.447).

**Conclusions:**

*FLNC* mutations were common in both HCM patients and healthy population. The pathogenicity of *FLNC* mutations detected in HCM patients and its association with the clinical outcomes should be cautiously interpreted.

## 1 INTRODUCTION

Hypertrophic cardiomyopathy (HCM) is a prevalent cardiac disease that affects about 1/500 of the total population (Maron et al., 1995; Zou et al., 2004). HCM has rather broad spectrums in both clinical manifestation and genetic etiology. Currently, HCM is considered to be an inherited disease, which presents an autosomal dominant trait. Previous studies have unveiled that several genes, mostly encoding sarcomere proteins, account for more than half of HCM cases (Authors/Task Force members et al., 2014). Recently, next‐generation sequencing provided convenient access to uncover the genetic causes for HCM, but have also produced a number of variants with uncertain significances in the disease.

*FLNC* (OMIM accession number 102,565) is the encoding gene of filamin C, an actin cross‐linking protein, widely expressed in cardiac and skeletal muscles (van der Flier & Sonnenberg, 2001). Mutations in *FLNC* were associated with myopathies, which mainly manifest skeletal muscle weakness (Fürst et al., 2013). Cardiac muscle involvement was considered to be an accessory manifestation to skeletal muscle damage in *FLNC*‐related myopathies. Recently, several studies reported that *FLNC* mutations are primary causes of HCM, dilated cardiomyopathy, and restrictive cardiomyopathy (Brodehl et al., 2016; Golbus et al., 2014; Valdés‐Mas et al., 2014). Valdes‐Mas et al. reported that several missense mutations of *FLNC* caused HCM. The patients affected by *FLNC* mutations had a higher probability of sudden cardiac death (SCD) (Valdés‐Mas et al., 2014). However, the pathogenicity and genotype–phenotype relationship of *FLNC* mutations need to be further evaluated. Herein, we comprehensively analyzed the mutation profile of the *FLNC* gene in an HCM cohort and healthy controls and investigated its association with the phenotypic expressions of the disease.

## 2 METHODS

### 2.1 Ethical compliance

The study was approved by The Ethics Committee of Fuwai Hospital. All the participants signed informed consent.

### 2.2 Study population

From 1999 to 2010, 540 unrelated HCM patients were enrolled into the present study. Diagnostic criteria of HCM were consistent with previous publications, mainly including a maximum wall thickness ≥15 mm in one or more left ventricle (LV) myocardial segments, which was not solely explained by abnormal loading conditions. Patients with long‐time history of hypertension were excluded when HCM group were enrolled. For patients with new‐onset arterial hypertension, diagnosis was made after systemic evaluation of imaging and electrocardiographic presentation, clinical manifestation, and family history. In addition, 307 healthy individuals were enrolled as controls. There were no cardiac or other systemic diseases found in any of the controls after a physical examination, 12‐lead electrocardiography, and echocardiography.

### 2.3 Genetic analysis

The coding exons and their flanking intronic regions of the *FLNC* gene were analyzed by targeted resequencing in all patients and control subjects, as described previously (Wang et al., 2014). Briefly, a sequencing library was constructed using peripheral blood genomic DNA, and the target regions were enriched with a custom‐designed probe library (Agilent Technologies, Santa Clara, CA, USA). The enriched fragment was sequenced with Illumina next‐generation sequencing platform (Illumina Inc., CA, USA). Sequencing reads were aligned with a human reference genome, and variant calling was performed. GenBank NM_001458.4 was adopted as the reference sequence.

To filter out common single nucleotide polymorphisms and neutral variants, variants with a minor allele frequency >1‰, in either the total population or in a Chinese population in the Exome Sequencing Project (ESP) database, 1,000 genomes database, and the ExAC database, were excluded from further analyses. All mutations included in the study were subsequently validated using Sanger sequencing.

The effect of missense variants on protein function was predicted with PolyPhen‐2, SIFT, and MutationTaster. Variants predicted as deleterious, by at least two algorithms, were considered to be pathogenic mutations. The effect of intronic variants on splicing was predicted with the Human Splicing Finder 3.0 algorithm.

### 2.4 Clinical data collection and follow‐up

Baseline data, including demographic characteristics, disease history, and examination results, were collected when patients were enrolled. Follow‐up was performed annually. The primary endpoint was all‐cause death. Cardiac mortality was defined as all deaths related to cardiovascular causes, including SCD, heart failure, and stroke. In this study, heart failure was defined as progression into NYHA 3–4 and acute congestive heart failure, which was different from the end‐stage heart failure requiring heart transplant. SCD was defined as unexpected death due to cardiac causes that occurred within 1 hr of symptom onset in a person with a known or unknown cardiac disease, or nocturnal death, with no antecedent history of worsening of symptoms. Sudden cardiac arrest (SCA) events included SCD, appropriate implantable cardiac defibrillator discharge, and defibrillated ventricular fibrillation. Heart failure events included acute heart failure symptom onset and chronic stepping into New York Heart Association (NYHA) functional class III/IV.

### 2.5 Statistics

Continuous variables were provided as mean ±standard deviation and compared with Student's *t* test or median (interquartile range) compared with Mann–Whitney *U* test. Categorical data were compared through a chi‐square test. Survival curves were constructed according to the Kaplan–Meier method, and comparisons were performed using the log‐rank test. All reported probability values were two‐tailed, and a *p*‐value <0.05 was considered statistically significant. SPSS 22.0 Statistical Software (SPSS, Chicago, IL, USA) and Prism GraphPad 5.0 (GraphPad Software, CA, USA) were used for calculations and illustrations.

## 3 RESULTS

### 3.1 Mutation profile of the FLNC gene

**Figure 1:**
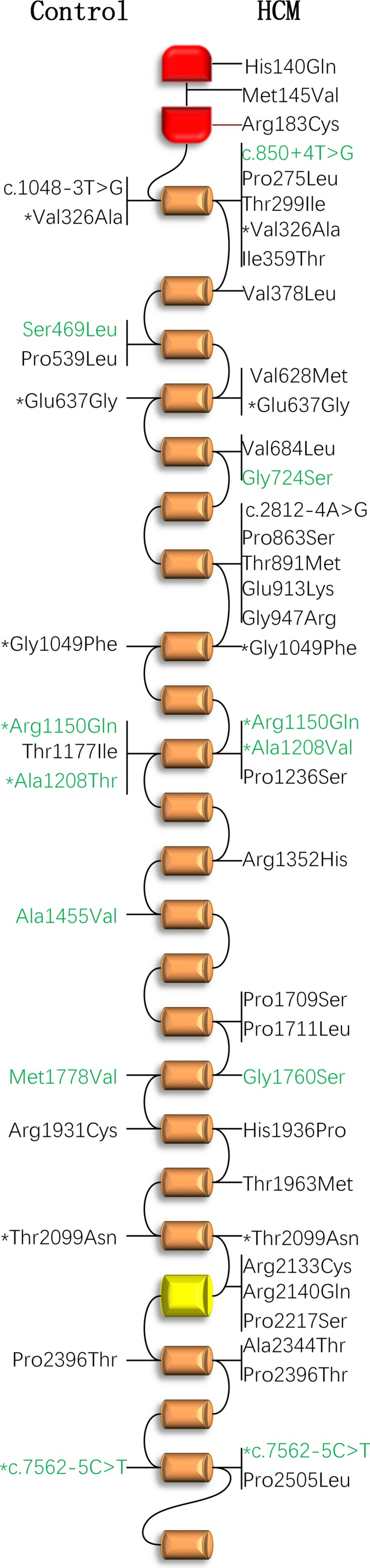
FLNC mutations and their protein positions. Distributions of FLNC mutations identified in patients with hypertrophic cardiomyopathy (up) and healthy controls (down) were showed. Green color indicates benign mutations suggested by bioinformatics prediction. * indicates the mutations detected in both patients and controls

In total, 43 mutations Figure 1 and Table 1, including 39 missense and four splicing, and four single‐nucleotide polymorphisms Table 2 were detected in the HCM cohort and control group. There were no nonsense and Insertion–Deletion mutations detected. Of these mutations, 34 were identified in 39 (7.22%) HCM patients, and 15 mutations were in 13 (4.23%) controls. There was no difference in the prevalence of *FLNC* mutations between patients and controls (*p* = 0.101). As shown in Figure 1, mutations found in patients and controls had a similar distribution among the functional domains of *FLNC*. Moreover, in the 39 patients with *FLNC* mutations, 13 (33.33%) harbored disease‐causing mutations in sarcomere genes, including four with MYH7 (OMIM accession number 160,760) mutations and nine with MYBPC3 (OMIM accession number 600,958) mutations.

**Table 1:** Detailed information of FLNC mutations identified in patients with hypertrophic cardiomyopathy and healthy controls

Mutation	Amino acid alteration	HCM	Control	ESP	1,000 genomes	ExAC	Protein domain	Polyphen−2	SIFT	Mutation Taster	Human Splicing Finder
c.420C>G	His140Gln	1 (1)	0	/	/	/	Actin‐binding	probably	affected	disease causing	N/A
c.433A>G	Met145Val	1(1)	0	/	/	/	Actin‐binding	benign	affected	disease causing	N/A
c.547C>T	Arg183Cys	1(1)	0	0.08‰	/	0.03‰	Actin‐binding	probably	affected	disease causing	N/A
c.824C>T	Pro275Leu	1(1)	0	/	/	/	Filamin 1	probably	affected	disease causing	N/A
c.896C>T	Thr299Ile	1(1)	0	/	/	0.01‰	Filamin 1	possibly	tolerated	disease causing	N/A
c.977 T>C	Val326Ala	1	1	/	0.20‰	0.07‰	Filamin 1	benign	affected	disease causing	N/A
c.1076 T>C	Ile359Thr	1	0	/	/	/	Filamin 1	probably	affected	disease causing	N/A
c.1132G>T	Val378Leu	1	0	/	/	/	Filamin 2	probably	affected	disease causing	N/A
c.1406C>T	Ser469Leu	0	1	/	/	/	Filamin 3	benign	affected	polymorphism	N/A
c.1616C>T	Pro539Leu	0	1	/	/	0.02‰	Filamin 3	probably	affected	disease causing	N/A
c.1882G>A	Val628Met	1	0	/	/	/	Filamin 4	probably	affected	disease causing	N/A
c.1910A>G	Glu637Gly	2	1	/	0.20‰	0.03‰	Filamin 4	possibly	affected	disease causing	N/A
c.2050G>C	Val684Leu	1	0	/	/	0.02‰	Filamin 5	benign	affected	disease causing	N/A
c.2170G>A	Gly724Ser	1	0	/	/	/	Filamin 5	benign	tolerated	disease causing	N/A
c.2587C>T	Pro863Ser	1	0	/	/	0.04‰	Filamin 7	possibly	affected	disease causing	N/A
c.2672C>T	Thr891Met	1(1)	0	/	/	0.03‰	Filamin 7	probably	affected	disease causing	N/A
c.2737G>A	Glu913Lys	1	0	/	/	0.01‰	Filamin 7	benign	tolerated	disease causing	N/A
c.2839G>C	Gly947Arg	1 (1)	0	/	/	0.15‰	Filamin 7	probably	affected	disease causing	N/A
c.3145G>T, c.3146G>T	Gly1049Phe	1 (1)	1	/	/	/	Filamin 8	probably	affected	disease causing	N/A
c.3449G>A	Arg1150Gln	1	1	0.08‰	0.60‰	0.07‰	Filamin 10	benign	tolerated	polymorphism	N/A
c.3530C>T	Thr1177Ile	0	1	/	/	/	Filamin 10	possibly	tolerated	disease causing	N/A
c.3622G>A	Ala1208Thr	0	1	/	0.40‰	0.02‰	Filamin 10	benign	tolerated	polymorphism	N/A
c.3623C>T	Ala1208Val	1	0	0.15‰	/	0.12‰	Filamin 10	benign	tolerated	polymorphism	N/A
c.3706C>T	Pro1236Ser	3	0	/	/	0.02‰	Filamin 10	probably	tolerated	disease causing	N/A
c.4364C>T	Ala1455Val	0	1	/	/	/	Filamin 13	benign	tolerated	polymorphism	N/A
c.5125C>T	Pro1709Ser	1	0	/	/	/	Filamin 15	benign	affected	disease causing	N/A
c.5132C>T	Pro1711Leu	1	0	/	/	0.02‰	Filamin 15	probably	affected	disease causing	N/A
c.5278G>A	Gly1760Ser	1 (1)	0	0.08‰	0.4‰	0.33‰	Filamin 16	benign	tolerated	polymorphism	N/A
c.5332A>G	Met1778Val	0	1	/	/	/	Filamin 16	benign	tolerated	polymorphism	N/A
c.5791C>T	Arg1931Cys	0	1	/	/	0.05‰	Filamin 17	probably	affected	disease causing	N/A
c.5807A>C	His1936Pro	1 (1)	0	/	/	/	Filamin 17	probably	affected	disease causing	N/A
c.5888C>T	Thr1963Met	1	0	/	/	0.02‰	Filamin 18	possibly	affected	disease causing	N/A
c.6296C>A	Thr2099Asn	4 (1)	1	/	/	0.01‰	Filamin 19	possibly	affected	disease causing	N/A
c.6397C>T	Arg2133Cys	1	0	/	/	/	Filamin 19	probably	affected	disease causing	N/A
c.6419G>A	Arg2140Gln	1 (1)	0	0.08‰	/	0.02‰	Filamin 19	probably	affected	disease causing	N/A
c.6649C>T	Pro2217Ser	1 (1)	0	/	/	/	Intradomain insert	benign	affected	disease causing	N/A
c.7030G>A	Ala2344Thr	1	0	/	0.40‰	0.02‰	Filamin 21	probably	affected	disease causing	N/A
c.7186C>A	Pro2396Thr	0	1	/	/	/	Filamin 21	probably	affected	disease causing	N/A
c.7514C>T	Pro2505Leu	1	0	/	/	/	Filamin 23	possibly	affected	disease causing	N/A
c.850 + 4 T>G	Splicing	1	0	/	/	0.02‰	Filamin 1	N/A			Benign
c.1048–3 T>G	Splicing	0	1	/	/	/	Filamin 1	N/A			Damaging
c.2812–4A>G	Splicing	1	0	/	/	/	Filamin 7	N/A			Damaging
c.7562–5C>T	Splicing	2	1	/	/	0.12‰	Filamin 23	N/A			Benign

Note

/indicates not detected; numbers in brackets indicate patients harboring other causative mutation; *N*/A, not available. Reference sequence, NM_001458.4.

**Table 2:** Detailed information of FLNC SNPs identified in patients with hypertrophic cardiomyopathy and healthy controls

SNP	Amino acid alteration	HCM	Control	ESP	1,000 genomes	ExAC	Protein domain	Polyphen−2	SIFT	Mutation Taster	Human splicing Finder 3.0
c.2686G>A	Gly896Arg	3	1	/	1‰	0.33‰	Filamin 7	probably	affected	disease causing	*N*/A
c.3079C>T	Arg1027Cys	1 (1)	0	/	4.9‰*	/	Filamin 8	probably	affected	disease causing	*N*/A
c.5764G>A	Ala1922Thr	5 (2)	3	0.08‰	1.8‰	1.37%	Filamin 17	benign	affected	disease causing	*N*/A
c.3790 + 5G>A	Splicing	1	0	0.31‰	4.9‰*	0.24‰	Filamin 11	N/A			Damaging

Note

SNP: single‐nucleotide polymorphism; / indicates not detected; * indicates frequency in Chinese population; numbers in brackets indicate patients harboring other causative mutation; *N*/A, Not Available. Reference sequence, NM_001458.4.

**Figure 2:**
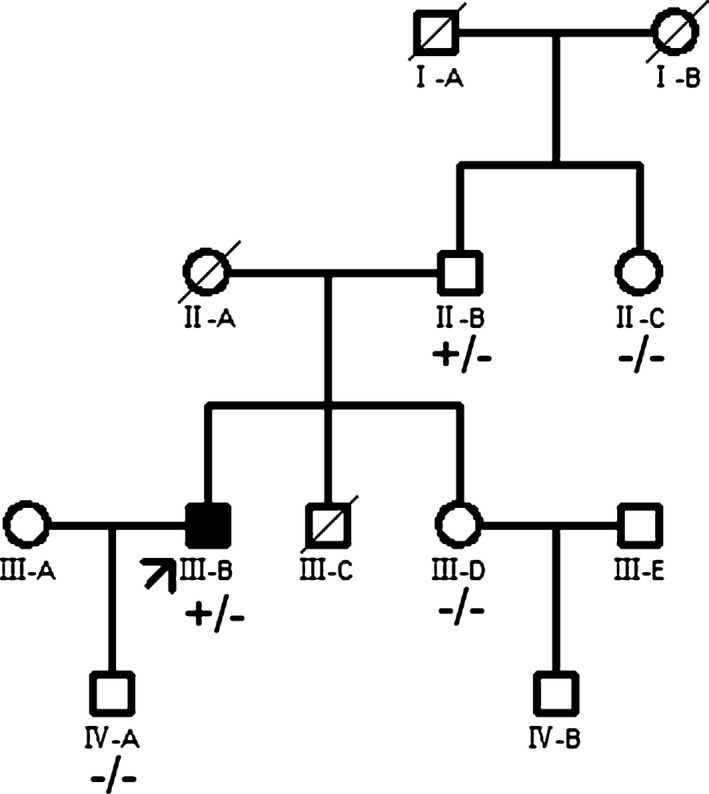
Pedigree analysis of Arg2133Cys mutation. Closed symbols indicate members with HCM phenotypes; open symbols denote non‐HCM members. The proband is denoted by an arrow. Circles indicate women, and squares refer to men. Slashed symbols indicate deceased members. ±, carrier of heterozygous mutation; ‐/‐, wild‐type

Bioinformatic analysis showed that 34 (79.07%) of the 43 mutations were deleterious, including 27 from 31 (5.74%) patients and nine from nine (2.93%) controls. Consistent with the total mutations, the deleterious mutations had a comparable prevalence and similar distribution in the functional domains among patient cohort and controls.

p.Arg2133Cys mutation was reported to cause HCM by previous study (Valdés‐Mas et al., 2014). We performed pedigree analysis for this variant Figure 2. p.Arg2133Cys was detected in proband's 83‐year‐old father (II‐B). II‐B had a septal thickness of 12 mm (posterior wall thickness of 8 mm). He didn't have systolic anterior motion of mitral valve or left ventricular outflow tract obstruction. His sister II‐C has a septal thickness of 11 mm (posterior wall thickness of 8 mm), without p.Arg2133Cys mutation. II‐B had a son (III‐C) who prematurely died because of developmental defect of brain.

### 3.2 Genotype–phenotype relationship

Baseline characteristics of the studied patients with HCM are listed in Table 3. There was no difference in the clinical expression observed at enrollment between patients, with and without *FLNC* mutations.

**Table 3:** Baseline characteristics of hypertrophic cardiomyopathy patients with and without FLNC mutations

Parameters	Total (*n* = 540)	With mutations (*n* = 39)	Without mutations (*n* = 501)	*p*‐value
Male (%)	376 (69.6)	23 (59.0)	353 (70.5)	0.149
Age (years)	50.1 ± 14.5	53.1 ± 16.9	49.8 ± 14.3	0.183
Height (cm)	166.8 ± 11.2	166.3 ± 8.2	166.8 ± 11.4	0.792
Weight (Kg)	71.4 ± 12.7	69.3 ± 9.8	71.6 ± 12.9	0.200
Heart rate (beats per minute)	72.4 ± 33.4	71.0 ± 12.2	72.5 ± 34.5	0.780
Systolic pressure (mmHg)	121.8 ± 17.8	122.9 ± 20.9	121.7 ± 17.6	0.673
Diastolic pressure (mmHg)	74.7 ± 10.8	74.4 ± 12.8	74.8 ± 10.6	0.862
Onset age (years)	42.9 ± 14.8	45.1 ± 16.9	42.8 ± 14.7	0.346
NYHA heart function class	1.66 ± 0.75	1.79 ± 0.74	1.65 ± 0.75	0.278
LVEDD (mm)	45.0 ± 6.7	43.9 ± 6.1	45.1 ± 6.7	0.303
LVEF (%)	66.2 ± 9.9	67.8 ± 8.1	66.0 ± 10.0	0.311
Left atrium (mm)	40.3 ± 6.9	38.3 ± 5.4	40.4 ± 7.1	0.084
Right ventricle (mm)	20.1 ± 3.6	20.1 ± 3.0	20.1 ± 3.6	0.970
Septal thickness (mm)	19.1 ± 5.9	20.7 ± 6.8	19.0 ± 5.8	0.080
Posterior wall thickness (mm)	11.8 ± 3.4	11.2 ± 2.6	11.8 ± 3.5	0.293
LVOT obstruction (%)	191 (35.4)	10 (25.6)	181 (36.1)	0.225
Family history (%)	135 (25.0)	9 (23.1)	126 (25.1)	0.850
Familial history of SCD (%)	80 (14.8)	6 (15.4)	74 (14.8)	0.819
Beta blocker	463 (85.7)	31 (79.5)	432 (86.2)	<0.001
Calcium channel blocker	259 (48.0)	29 (74.4)	230 (45.9)	<0.001
Prior or future SRT	98 (18.1)	5 (12.8)	93 (18.6)	<0.001

Note

NYHA: New York Heart Association; LVEDD: left ventricular end‐diastolic diameter; LVEF: left ventricular eject fraction; LVOT: left ventricular outflow tract; SCD: sudden cardiac death; SRT: septal reduction therapy.

**Figure 3:**
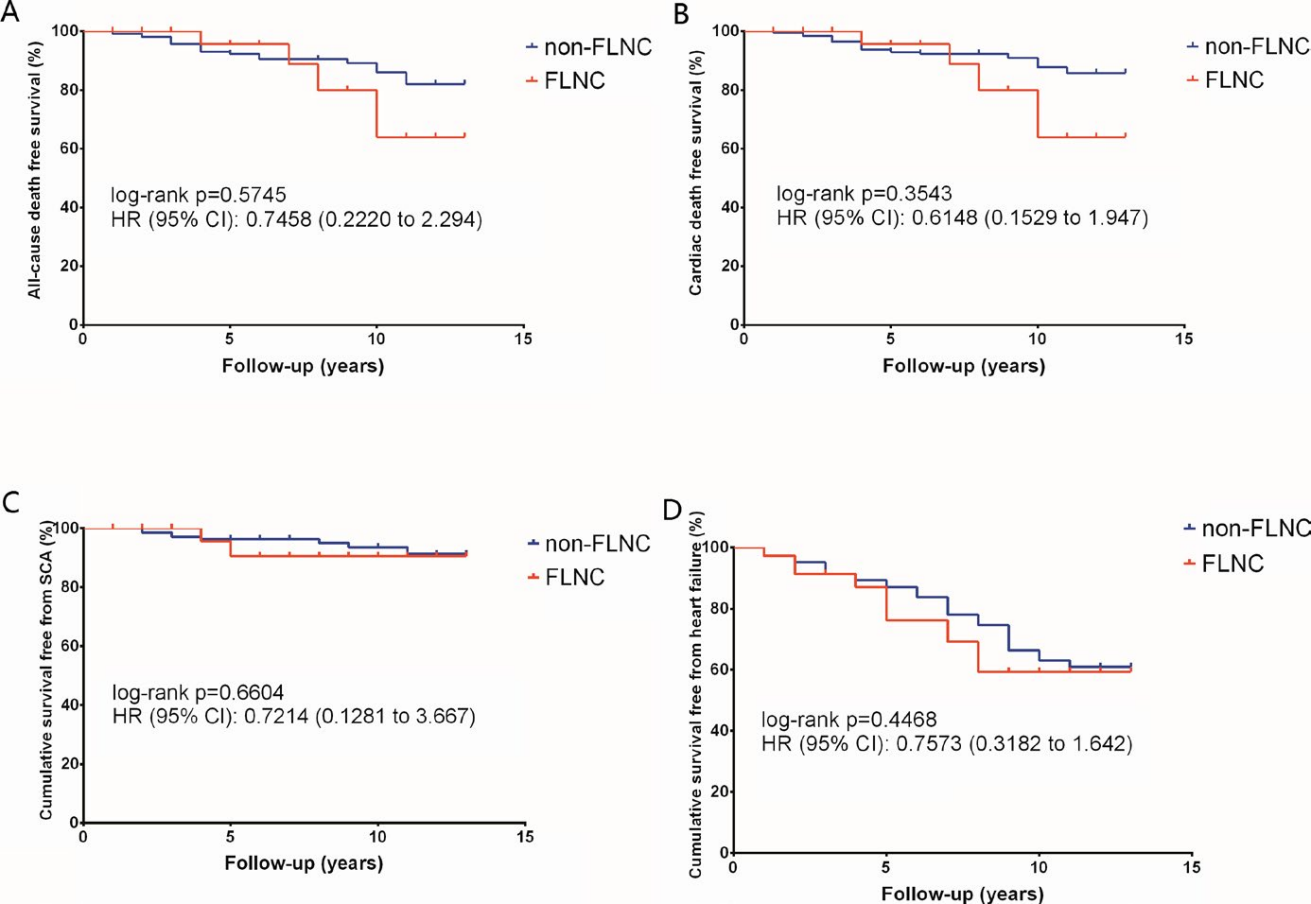
Survival curves in hypertrophic cardiomyopathy patients with and without FLNC mutations. *p*‐values were calculated using the log‐rank test. FLNC mutations did not increase the risk for either all‐cause mortality (a) or cardiac mortality (b); there was no significant difference in survival, free from sudden cardiac arrest (c) and heart failure (d), between HCM patients, with and without FLNC mutations

During the follow‐up of 4.7 ± 3.2 years, 45 patients had died, including 38 from cardiac mortality and four from cancer. Four patients with *FLNC* mutations died from cardiac mortality and were of a similar age to the 34 *FLNC* mutation‐negative patients that died from cardiac mortality (63.00 ± 29.47 vs. 49.71 ± 18.13 years old, *p* = 0.202). The cause of death in patients with *FLNC* mutations included one SCD and three heart failures Table 4. Survival curve analysis showed that the patients with and without *FLNC* mutations had similar risks for both all‐cause mortality (HR 0.746, 95% CI 0.222–2.295, *p* = 0.575) (A, Figure 3 and cardiac mortality (HR 0.615, 95% CI 0.153–1.947, *p* = 0.354) (B, Figure 3. Moreover, patients with *FLNC* mutations had a comparable risk of SCA (HR 0.721, 95% CI 0.128–3.667, *p* = 0.660) (C, Figure 3 and heart failure (HR 0.757, 95% CI 0.318–1.642, *p* = 0.447) (D, Figure 3 to patients without mutations.

**Table 4:** Died patients with FLNC mutations

No.	Cause of death	Gender	Age at death	FLNC mutation	Concomitant mutation	NYHA class	Family history of SCD
1	HF	F	84	c.2812–4A>G	No	2	No
2	HF	F	83	p.Arg2140Gln	MYBPC3	4	No
3	SCD	M	64	p.Pro2217Ser	MYBPC3	1	Yes
4	HF	F	21	p.Thr2099Asn	No	3	No

Note

SCD: sudden cardiac death; HF: heart failure; F: female; M: male; NYHA, New York Heart Association

Similar results were also observed in the clinical outcomes in patients with and without deleterious *FLNC* mutations. Deleterious mutations did not significantly increase the risk of all‐cause mortality (HR 0.6752, 95% CI 0.1534–2.514, *p* = 0.5077), cardiac mortality (HR 0.5595, 95% CI 0.1013–2.133, *p* = 0.3279), SCA (HR 0.4865, 95% CI 0.05021–2.673, *p* = 0.3241), and heart failure (HR 0.5619, 95% CI 0.1699–1.244, *p* = 0.1332), respectively. Furthermore, no patients with *FLNC* mutations presented skeletal muscle dysfunction at baseline or developed skeletal muscle lesion during follow‐up.

## 4 DISCUSSION

HCM is a monogenic cardiac disease caused by mutations in a variety of genes. Genetic testing of these disease genes in probands, and of which families have been demonstrated to be useful in diagnosis, and sometime in risk stratification (Authors/Task Force members et al., 2014; Gersh et al., 2011). Disease‐causing mutations can be identified in about half of the patients with HCM, mostly located in the genes encoding sarcomere proteins. The pathogenicity of mutations in genes beyond sarcomere is usually less certain (Seidman & Seidman, 2011), which lessened its clinical usefulness. Recently, mutations in the *FLNC* gene were reported to cause HCM and were also reported to be related to a high risk of SCD (Valdés‐Mas et al., 2014). However, mutation profile analysis revealed that *FLNC* mutation was common in both HCM and healthy populations, and lacked association with clinical expressions and prognosis in patients with HCM.

Filamin C is primarily expressed in striated muscles and plays roles in muscular contraction through interaction with Z‐disc and sarcolemma. *FLNC* mutation was initially found to cause myopathy in a myofibrillar myopathy family, caused by a hotspot nonsense mutation p.W2710X (Vorgerd et al., 2005). Since then, several other disease mutations in familial myopathy patients have been unveiled (Avila‐Smirnow, 2010; Duff et al., 2011; Guergueltcheva et al., 2011; Kley et al., 2007; Luan, Hong, Zhang, Wang, & Yuan, 2010; Shatunov et al., 2009; Tasca et al., 2012). Only a few patients in these reports exhibited myocardial involvement. Therefore, *FLNC* has long been considered as a disease gene of primary skeletal myopathy, rarely accompanied by myocardial abnormality.

Recently, mutations in *FLNC* genes were posited to induce various cardiomyopathies (Brodehl et al., 2016; Golbus et al., 2014), including HCM (Valdés‐Mas et al., 2014). Eleven missense mutations of *FLNC* were identified in familial and sporadic patients with HCM (Jaafar et al., 2016; Valdés‐Mas et al., 2014). These mutations are distributed in various domains of the filamin C protein. To clarify its mutation profile, the present study comprehensively sequenced *FLNC* genes in an HCM patient cohort and healthy controls. We found that *FLNC* mutations were not rare, even in healthy controls, with a frequency of about 4%. It is far higher than HCM prevalence (0.2%) in general population. This strongly implicates that most mutations do not lead to HCM. The mutations of *FLNC* were not significantly enriched in HCM patients. Gomez et al conducted a similar study in which they used unreported variants to compare the difference of mutation prevalence between HCM and healthy group (Gómez et al., 2017). If all variants in their study were included, the prevalence (38 variants in 448 HCM patients and 22 variants in 450 healthy controls) would be similar to our data (34 variants in 540 HCM patients and 15 variants in 307 healthy controls). Furthermore, about one third of the patients with *FLNC* mutations also carried pathogenic mutations in genes encoding for sarcomere proteins. The prevalence of sarcomere mutations in patients with FNLC mutations was not apparently lower than that previously reported in cohorts from China and other countries (Jensen et al., 2013; Morita et al., 2008; Wang et al., 2014). These results indicated that the pathogenicity of a significant proportion of mutations identified in *FLNC* gene might be uncertain for HCM. However, considering the relatively small study volume, further studies are needed to validate the prevalence.

*FLNC* was recently to be associated with multiple types of cardiomyopathy including hypertrophic cardiomyopathy (Valdés‐Mas et al., 2014), restrictive cardiomyopathy (Brodehl et al., 2016), dilated cardiomyopathy (Reinstein et al., 2016), and arrhythmogenic cardiomyopathy (Ortiz‐Genga et al., 2016). More interestingly, *FLNC*‐cardiomyopathy patients rarely had skeletal myopathy, which is another pathological result of *FLNC* mutation. Previous study showed that non‐genetic factors play important role in HCM phenotype in MYL2 (OMIM accession number 160,781) mutation carriers (Claes et al., 2016). Considering the high incidence and wide distribution of *FLNC* mutation, other factors may play crucial role in deciding phenotype.

The genotype–phenotype relationship has great significance for risk stratification in patients with HCM. Early studies suggested the located genes or the type of sarcomere mutations were related to clinical outcomes of patients with HCM, but these relationships were subsequently demonstrated to be greatly varied. Also, it is difficult to define the pathogenicity when a mutation is detected. Though pedigree analysis is a useful method, it may not work well for sporadic carriers and late‐onset disease. Moreover, bias may be introduced when the causative mutation is identified with pedigree analysis. It may exclude less phenotyped individuals who have milder symptom and better outcome. An overall evaluation of all probably causative mutations could supply more implications to clinicians in genetic screening. In the present study, the baseline characteristics of patients with *FLNC* mutations were similar to those without *FLNC* mutations. During follow‐up, only one patient died from SCD at 63 years old. SCD risk in HCM patients, with and without *FLNC* mutations, was comparable. This was different to the study by Valdes‐Mas et al because the compared the SCD risk in ascertained HCM patients with causative *FLNC* mutation. However, SCD risk in all mutation carriers including phenotype‐negative carriers was not available. In contrast, our data suggested low SCD risk of common mutations with uncertain clinical significance. We also observed no effect of *FLNC* mutations on other clinical outcomes, including cardiac and all‐cause mortalities, and progression to heart failure. The present study suggested that the genotype–phenotype relationship of *FLNC* mutations might be uncertain and need to be further evaluated.

Our study has several limitations. First, all patients were recruited from a single center, which might introduce selection bias. Second, the lack of enough pedigree analysis led to a lack of pathogenic evaluation of each individual mutation identified in *FLNC*. It made us unable to investigate the characteristic of disease‐causing mutations. Third, all patients in the present study were from the Chinese Han population, and the pathogenicity and clinical relevance of *FLNC* mutations in patients with HCM remain to be evaluated in other people.

In conclusion, our study found that *FLNC* mutation was relatively common in both HCM patients and healthy population. Patients with and without FNLC mutations had comparable clinical outcomes. The significance of identified *FLNC* mutations in patients with HCM should be cautiously interpreted in genetic testing.

## CONFLICT OF INTEREST

The authors have no disclosures.

## References

[mgg3488-bib-0001] Avila‐Smirnow, D. (2010). P2.18 A novel missense FLNC mutation causes arrhythmia and late onset myofibrillar myopathy with particular histopathology features. Neuromuscular Disorders, 20, 623–624. 10.1016/j.nmd.2010.07.090

[mgg3488-bib-0002] Brodehl, A. , Ferrier, R. A. , Hamilton, S. J. , Greenway, S. C. , Brundler, M. A. , Yu, W. , … Gerull, B. (2016). Mutations in FLNC are associated with familial restrictive cardiomyopathy. Human Mutatation, 37, 269–279. 10.1002/humu.22942 26666891

[mgg3488-bib-0003] Claes, G. R. , van Tienen, F. H. , Lindsey, P. , Krapels, I. P. , Helderman‐van den Enden, A. T. , Hoos, M. B. , ... van den Wijngaard, A. (2016). Hypertrophic remodelling in cardiac regulatory myosin light chain (MYL2) founder mutation carriers. European Heart Journal, 37, 1815–1822. 26497160 10.1093/eurheartj/ehv522

[mgg3488-bib-0004] Duff, R. M. , Tay, V. , Hackman, P. , Ravenscroft, G. , McLean, C. , Kennedy, P. , … Laing, N. G. (2011). Mutations in the N‐terminal actin‐binding domain of filamin C cause a distal myopathy. American Journal of Human Genetics, 88, 729–740. 10.1016/j.ajhg.2011.04.021 21620354 PMC3113346

[mgg3488-bib-0005] Authors/Task Force members , Elliott, P. M. , Anastasakis, A. , Borger, M. A. , Borggrefe, M. , Cecchi, F. , … Watkins, H. (2014). ESC Guidelines on diagnosis and management of hypertrophic cardiomyopathy: The Task Force for the Diagnosis and Management of Hypertrophic Cardiomyopathy of the European Society of Cardiology (ESC). European Heart Journal, 35, 2733–2779. 25173338 10.1093/eurheartj/ehu284

[mgg3488-bib-0006] Fürst, D. O. , Goldfarb, L. G. , Kley, R. A. , Vorgerd, M. , Olivé, M. , & van der Ven, P. F. (2013). Filamin C‐related myopathies: Pathology and mechanisms. Acta Neuropathologica, 125, 33–46. 23109048 10.1007/s00401-012-1054-9 PMC5127197

[mgg3488-bib-0007] Gersh, B. J. , Maron, B. J. , Bonow, R. O. , Dearani, J. A. , Fifer, M. A. , Link, M. S. , … Yancy, C. W. (2011). 2011 ACCF/AHA guideline for the diagnosis and treatment of hypertrophic cardiomyopathy: A report of the American College of Cardiology Foundation/American Heart Association Task Force on Practice Guidelines. Circulation, 124, e783–e831. 22068434 10.1161/CIR.0b013e318223e2bd

[mgg3488-bib-0008] Golbus, J. R. , Puckelwartz, M. J. , Dellefave‐Castillo, L. , Fahrenbach, J. P. , Nelakuditi, V. , Pesce, L. L. , … Mcnally, E. M. (2014). Targeted analysis of whole genome sequence data to diagnose genetic cardiomyopathy. Circulation Cardiovascular Genetics, 7, 751–759. 10.1161/CIRCGENETICS.113.000578 25179549 PMC4270910

[mgg3488-bib-0009] Gómez, J. , Lorca, R. , Reguero, J. R. , Morís, C. , Martín, M. , Tranche, S. , … Cote, E. (2017). Screening of the filamin C gene in a large cohort of hypertrophic cardiomyopathy patients. Circulation Cardiovascular Genetics, 10 10.1161/CIRCGENETICS.116.001584 28356264

[mgg3488-bib-0010] Guergueltcheva, V. , Peeters, K. , Baets, J. , Ceuterick‐de Groote, C. , Martin, J. J. , Suls, A. , … Jordanova, A. (2011). Distal myopathy with upper limb predominance caused by filamin C haploinsufficiency. Neurology, 77, 2105–2114. 10.1212/WNL.0b013e31823dc51e 22131542

[mgg3488-bib-0011] Jaafar, N. , Gómez, J. , Kammoun, I. , Zairi, I. , Amara, W. B. , Kachboura, S. , … Coto, E. (2016). Spectrum of mutations in hypertrophic cardiomyopathy genes among Tunisian patients. Genetic Testing and Molecular Biomarkers, 20, 674–679. 27574918 10.1089/gtmb.2016.0187

[mgg3488-bib-0012] Jensen, M. K. , Havndrup, O. , Christiansen, M. , Andersen, P. S. , Diness, B. , Axelsson, A. , … Bundgaard, H. (2013). Penetrance of hypertrophic cardiomyopathy in children and adolescents: A 12‐year follow‐up study of clinical screening and predictive genetic testing. Circulation, 127, 48–54. 10.1161/CIRCULATIONAHA.111.090514 23197161

[mgg3488-bib-0013] Kley, R. A. , Hellenbroich, Y. , van der Ven, P. F. , Fürst, D. O. , Huebner, A. , Bruchertseifer, V. , ... Vorgerd, M. (2007). Clinical and morphological phenotype of the filamin myopathy: A study of 31 German patients. Brain, 130, 3250–3264. 10.1093/brain/awm271 18055494

[mgg3488-bib-0014] Luan, X. , Hong, D. , Zhang, W. , Wang, Z. , & Yuan, Y. (2010). A novel heterozygous deletion‐insertion mutation (2695–2712 del/GTTTGT ins) in exon 18 of the filamin C gene causes filaminopathy in a large Chinese family. Neuromuscular Disorders, 20, 390–396. 10.1016/j.nmd.2010.03.009 20417099

[mgg3488-bib-0015] Maron, B. J. , Gardin, J. M. , Flack, J. M. , Gidding, S. S. , Kurosaki, T. T. , & Bild, D. E. (1995). Prevalence of hypertrophic cardiomyopathy in a general population of young adults. Echocardiographic analysis of 4111 subjects in the CARDIA Study. Coronary Artery Risk Development in (Young) Adults. Circulation, 92, 785–789. 10.1161/01.CIR.92.4.785 7641357

[mgg3488-bib-0016] Morita, H. , Rehm, H. L. , Menesses, A. , McDonough, B. , Roberts, A. E. , Kucherlapati, R. , … Seidman, C. E. (2008). Shared genetic causes of cardiac hypertrophy in children and adults. The New England Journal of Medicine, 358, 1899–1908. 10.1056/NEJMoa075463 18403758 PMC2752150

[mgg3488-bib-0017] Ortiz‐Genga, M. F. , Cuenca, S. , DalFerro, M. , Zorio, E. , Salgado‐Aranda, R. , Climent, V. , … Monserrat, L. (2016). Truncating FLNC mutations are associated with high‐risk dilated and arrhythmogenic cardiomyopathies. Journal of the American College of Cardiology, 68, 2440–2451. 10.1016/j.jacc.2016.09.927 27908349

[mgg3488-bib-0018] Reinstein, E. , Gutierrez‐Fernandez, A. , Tzur, S. , Bormans, C. , Marcu, S. , Tayeb‐Fligelman, E. , ... Lopez‐Otin, C. (2016). Congenital dilated cardiomyopathy caused by biallelic mutations in filamin C. European Journal of Human Genetics, 24, 1792–1796. 10.1038/ejhg.2016.110 27601210 PMC5117915

[mgg3488-bib-0019] Seidman, C. E. , & Seidman, J. G. (2011). Identifying sarcomere gene mutations in hypertrophic cardiomyopathy: A personal history. Circulation Research, 108, 743–750. 10.1161/CIRCRESAHA.110.223834 21415408 PMC3072749

[mgg3488-bib-0020] Shatunov, A. , Olivé, M. , Odgerel, Z. , Stadelmann‐Nessler, C. , Irlbacher, K. , van Landeghem, F. , … Goldfarb, L. G. (2009). In‐frame deletion in the seventh immunoglobulin‐like repeat of filamin C in a family with myofibrillar myopathy. European Journal of Human Genetics, 17, 656–663. 10.1038/ejhg.2008.226 19050726 PMC2672961

[mgg3488-bib-0021] Tasca, G. , Odgerel, Z. , Monforte, M. , Aurino, S. , Clarke, N. F. , Waddell, … Goldfarb, L. G. (2012). Novel FLNC mutation in a patient with myofibrillar myopathy in combination with late‐onset cerebellar ataxia. Muscle and Nerve, 46, 275–282. 10.1002/mus.23349 22806379 PMC3400116

[mgg3488-bib-0022] Valdés‐Mas, R. , Gutiérrez‐Fernández, A. , Gómez, J. , Coto, E. , Astudillo, A. , Puente, D. A. , … López‐Otín, C. (2014). Mutations in filamin C cause a new form of familial hypertrophic cardiomyopathy. Nature Communications, 5, 5326. 10.1038/ncomms6326 25351925

[mgg3488-bib-0023] van der Flier, A. , & Sonnenberg, A. (2001). Structural and functional aspects of filamins. Biochimica Et Biophysica Acta, 1538, 99–117. 10.1016/S0167-4889(01)00072-6 11336782

[mgg3488-bib-0024] Vorgerd, M. , van der Ven, P. F. , Bruchertseifer, V. , Löwe, T. , Kley, R. A. , Schröder, R. , ... Huebner, A. (2005). A mutation in the dimerization domain of filamin c causes a novel type of autosomal dominant myofibrillar myopathy. American Journal of Human Genetics, 77, 297–304. 10.1086/431959 15929027 PMC1224531

[mgg3488-bib-0025] Wang, J. , Wang, Y. , Zou, Y. , Sun, K. , Wang, Z. , Ding, H. , ... Song, L. (2014). Malignant effects of multiple rare variants in sarcomere genes on the prognosis of patients with hypertrophic cardiomyopathy. European Journal of Heart Failure, 16, 950–957. 10.1002/ejhf.144 25132132

[mgg3488-bib-0026] Zou, Y. , Song, L. , Wang, Z. , Ma, A. , Liu, T. , Gu, H. , ... … R. (2004). Prevalence of idiopathic hypertrophic cardiomyopathy in China: A population‐based echocardiographic analysis of 8080 adults. The American Journal of Medicine, 116, 14–18. 10.1016/j.amjmed.2003.05.009. 14706660

